# Application of functionalized nanofluid in thermosyphon

**DOI:** 10.1186/1556-276X-6-494

**Published:** 2011-08-16

**Authors:** Xue-Fei Yang, Zhen-Hua Liu

**Affiliations:** 1 School of Mechanical Engineering, Shanghai Jiaotong University, Shanghai 200240, People's Republic of China

**Keywords:** nanofluid, surface functionalization, thermosyphon, heat transfer

## Abstract

A water-based functionalized nanofluid was made by surface functionalizing the ordinary silica nanoparticles. The functionalized nanofluid can keep long-term stability. and no sedimentation was observed. The functionalized nanofluid as the working fluid is applied in a thermosyphon to understand the effect of this special nanofluid on the thermal performance of the thermosyphon. The experiment was carried out under steady operating pressures. The same work was also explored for traditional nanofluid (consisting of water and the same silica nanoparticles without functionalization) for comparison. Results indicate that a porous deposition layer exists on the heated surface of the evaporator during the operating process using traditional nanofluid; however, no coating layer exists for functionalized nanofluid. Functionalized nanofluid can enhance the evaporating heat transfer coefficient, while it has generally no effect on the maximum heat flux. Traditional nanofluid deteriorates the evaporating heat transfer coefficient but enhances the maximum heat flux. The existence of the deposition layer affects mainly the thermal performance, and no meaningful nanofluid effect is found in the present study.

## Introduction

The revolution of fabrication technology allows the fabrication of materials at a nano-scale. Nanoparticles fabricated by different methods show various fancy characteristics in electronic, magnetic, optical, and catalytic applications. The concept of the nanofluid, which is the suspension of nanoparticles, was firstly proposed by Choi [1]. Since then, a lot of researches have been carried out to study the heat transfer characteristics of nanofluids. The heat transfer characteristics of nanofluids started with the investigation of thermal conductivity [1-3], then the single-phase flow heat transfer [4-7], and now, the focus mainly is on the phase-changing heat transfer of nanofluids. Among the phase-changing heat transfer, the application of nanofluids in heat pipes gains increasing popularity [8-25]. The involved heat pipes include the grooved heat pipe [8,9], wicked heat pipe [10,11], sintered heat pipe [12,13], oscillated heat pipe [14,15], and the thermosyphon [16-25].

Xue *et al. *[16] studied the heat transfer performance of carbon nanotube-water nanofluid in a thermosyphon. The mass concentration of nanoparticles is 1.3158 wt.%. The thermosyphon is a copper tube with an outer diameter (O.D.) of 20 mm. The filling ratio is 20%. Results show that the thermosyphon with carbon nanotube nanofluid has a higher evaporation section wall temperature, incipience temperature, and excursion, as well as thermal resistance. The carbon nanotube-water nanofluid deteriorates the heat transfer of the thermosyphon compared with the water case.

Khandekar *et al. *[17,18] investigated the overall thermal resistance of a closed two-phase thermosyphon using water-based Al_2_O_3 _(40 to 47 nm), CuO (8.6 to 13.5 nm), and laponite clay (disks with a diameter of 25 nm and thickness of 1 nm) nanofluids. The length and the inner diameter of the thermosyphon are 720 and 16 mm, respectively. The nanoparticle mass concentration is 1.0 wt.%. Results show that all nanofluids have inferior thermal performance compared to pure water. A mechanism analysis guesses that the increase in wettability and entrapment of nanoparticles in the grooves of the surface cause a decrease of the Peclet number in the evaporator side and finally leads to poor thermal performance.

Naphon *et al. *[19] studied the heat transfer performance of the TiO_2 _-water and TiO_2_-alcohol nanofluids in a thermosyphon. The nanoparticle volume concentration is 0.01%, 0.05%, 0.10%, and 0.50%, respectively. The thermosyphon is made of a copper tube with an O.D. of 15 mm and a length of 600 mm. The authors investigated the effects of filling ratio, inclined angle, and volume concentration on the heat transfer performance. Results show that nanoparticles can enhance the heat transfer efficiency by 10.6%. Naphon *et al. *[20] also studied the heat transfer of TiO_2_-R11 nanofluid in a thermosyphon with the nanoparticle volume concentrations of 0.01%, 0.05%, 0.10%, 0.50%, and 1.0%. Results show that the thermosyphon efficiency can be enhanced by 40%.

Liu *et al. *[21,22] investigated the effect of nanoparticle parameters on the thermal performance in a thermosyphon using CuO and carbon nanotube nanofluids without surfactants. The diameter, the evaporator, the adiabatic section, and the condenser of the thermosyphon have a length of 8, 100, 100, and 150 mm, respectively. The experimental results show that adding nanoparticles in the heat pipe could enhance both the heat transfer performance of evaporation section and the maximum heat flux (MHF). Different from other studies, their experiments were carried out at several steady operating pressures, and the experiments found that the operation pressure has a significant influence on the heat transfer enhancement.

Noie *et al. *[23] studied the Al_2_O_3_-water nanofluid in a thermosyphon. The thermosyphon is made of a copper tube with an inner diameter of 20 mm and a length of 1,000 mm. The length of the evaporator and the condenser is 350 and 400 mm, respectively. The nanoparticle volume concentration is 1% to 3%. Results show that the nanofluid can enhance the heat pipe efficiency by 14.7%, and the thermosyphon shows a more uniformly distributed temperature.

Paramatthanuwat *et al. *[24] studied the heat transfer of Ag-water nanofluid in a thermosyphon. The effects of filling ratio (30%, 50%, 80%), the operating temperature (40°C, 50°C, 60°C), the ratio of length and diameter (5, 10, 20), and the diameter (7.5, 11.1, and 25.4 mm) on the heat transfer performance were investigated in detail. Results show that the heat transfer capacity can be enhanced by 70% by adding Ag nanoparticles.

Teng *et al. *[25] studied the heat transfer performance of the Al_2_O_3_-water nanofluid (mass concentrations of 0.5%, 1.0%, and 3.0%). The thermosyphon is made of a copper tube with an inner diameter of 8 mm and a length of 600 mm. The authors investigated the effects of inclination, filling ratio, and mass concentration on the heat transfer performance. The thermosyphon efficiency can be enhanced by 16.8% at the mass concentration of 1.0%.

Besides, the type and the preparation method of nanofluids can also lead to the difference of the heat transfer of a thermosyphon using nanofluids. Two ways are usually used to prepare nanofluids: the one-step method and the two-step method. The one-step method simultaneously makes and disperses nanoparticles into base fluids. The two-step method first produces the nanoparticles and then disperses nanoparticles in base fluids. The two-step method is more widely used because of its convenience, low cost, and large-amount producing capacity. Therefore, most of the literatures reported use the two-step method, but the stability of nanofluids prepared by the two-step method is a key issue preventing their commercial application. Nanoparticles tend to aggregate due to the van der Waals attraction. Nanoparticles will settle out of the base fluids if severe aggregation happens. The surface functionalization technique is a promising way to solve this problem. The current authors have reported a method to prepare a kind of functionalized nanofluid that have good stability for a long run [26]. The nanoparticles used were functionalized silica nanoparticles by grafting silanes to the surface of silica nanoparticles. After the surface functionalization process, nanofluids were prepared by the two-step method using functionalized nanoparticles and deionized water. Functionalized nanoparticles were dispersed into deionized water, and the solution was kept standing for 12 h with an environmental temperature of 50°C. Then well-dispersed nanofluid can be prepared without any surfactant used. Functionalized nanoparticles can still keep dispersing well after the nanofluid has been standing for 12 months, and no sedimentation was observed. The covalent bonding "Si-O-Si" helps maintain the steric stabilization effect formed by the grafting silanes which contribute to the long-term stability of the nanofluids.

On the other hand, for traditional nanofluids (prepared with nanoparticles without functionalization), a deposition layer usually forms on the heated surface during the phase-changing heat transfer. However, for functionalized nanofluid, no deposition layer forms on the heated surface during the phase-changing heat transfer process, which guarantees the stability and the reliability of the operating equipment using nanofluids as working fluids [26].

Based on the good stability and the no deposition feature of functionalized nanofluid, it is applied in a thermosyphon as the working fluid to improve the thermal performance of the thermosyphon in the present study. The main purpose is to investigate the sole effect of the thermophysical properties of nanofluids on the thermal performance of the thermosyphon under the condition that no coating layer exists on the smooth heated surface. The present work studied mainly the phase-change heat transfer characteristics including the evaporating and condensing heat transfer of functionalized nanofluid in a thermosyphon. The same work was also explored on traditional nanofluid for better understanding of the phase-change heat transfer mechanism of nanofluids in a thermosyphon. Nanoparticles used for traditional nanofluids are the same with those used for preparing functionalized nanoparticles. The experimental conditions are also the same. In addition, the surface characteristics of heated surfaces of functionalized nanofluid and traditional nanofluid after operating experiments are measured to judge the effect of heated surface on the thermal performance. The heat transfer mechanism of nanofluids is investigated and discussed in the present study.

## Experimental apparatus and process

**Figure 1:**
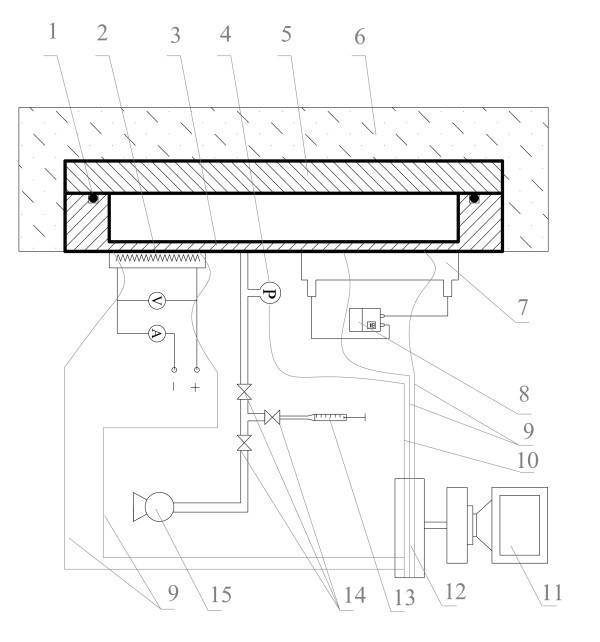
**Schematic of experimental apparatus**.

**Figure 2:**
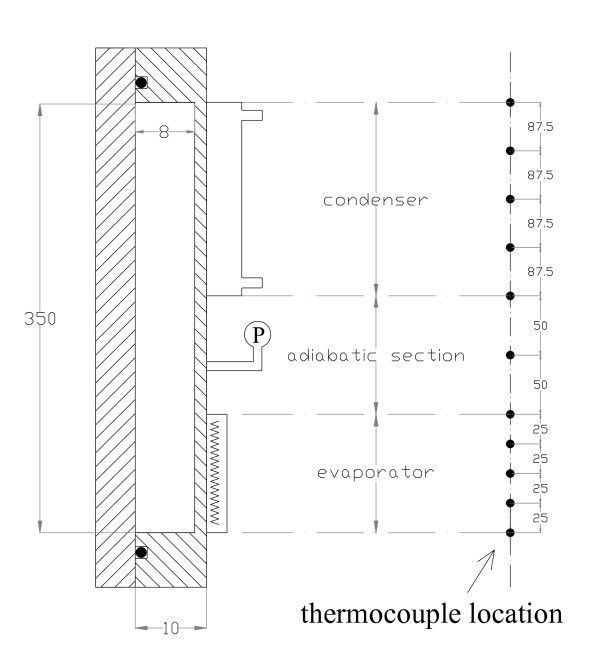
**Schematic of the thermosyphon (unit, millimeter)**.

A schematic view of the experimental apparatus is shown in Figure 1. It consisted of a rectangular plate thermosyphon made of copper, a heating system, a condensing system, a data acquisition system, a power supply, a vacuum pumping unit, and a liquid filling device. The rectangular thermosyphon shown in Figure 2 was vertically positioned with its inner chamber size of 350 × 100 × 8 mm. A Teflon cover was fixed together with the copper chamber and rubber O-ring for vacuum sealing. The lengths of the evaporator, the adiabatic section, and the condenser of the thermosyphon were 100, 100, and 150 mm, respectively. The hydraulic equivalent diameter of the channel was equal to the channel thickness (8 mm). The evaporator section was heated by a film heater connected to a power supply. The condenser section was cooled by cooling water circulating in a cooling jacket. Thirteen thermocouples were used to measure the system temperature including five of them for the temperatures of the evaporator wall, five for those of the condenser wall, two for those of the cooling water at the inlet and outlet, and one for that of the vapor in the thermosyphon. A pressure transducer measuring the system operating pressure was installed near the thermocouple measuring the vapor temperature.

The experiment was carried out at three steady operating pressures of 7.38, 15.75, and 31.18 kPa, which correspond to the operating temperatures (the vapor saturated temperatures) of 40°C, 55°C, and 70°C, respectively. The measured vapor temperature in the vapor line was taken as the operating temperature. Temperature and velocity of the cooling water were carefully controlled to keep the operating pressure at a constant value for varying heat fluxes. A data acquisition system was used to collect the digital signals of the thermocouples and the pressure transducer.

Before each test, the vacuum pumping process and liquid preheating process were performed to remove the gases dissolved in the thermosyphon. The vacuum pressure was pumped to be less than 8 × 10^-3 ^Pa to eliminate the influence of incondensable gases. Rationed nanofluid was filled into the thermosyphon through vacuum valves. The filling volume was kept at 25% of that of the thermosyphon, 87.5% of that of the evaporator. In the run, the heating power was gradually increased by an increment of 5%. When the measured wall temperature increased abruptly and could not hold a steady state, which indicated that a dry-out phenomenon occurred on the wall, the heating power supply was instantly switched off. Then, the run was restarted from the former steady heating power, and the power was then increased in an increment of 1% of the former power. When the measured wall temperatures once again increased abruptly and could not hold a steady state, the electric power supply was instantly switched off, and the test was stopped. The MHF value was determined from the heating power of the former time.

To investigate the surface morphology of the heated surface during the evaporating process, a polished copper sheet with an area of 10 × 10 mm was soldered to the inner surface of the evaporator and the condenser using soldering tin. The copper sheet was taken off after the experiment by melting the soldering tin. The scanning electron microscope (SEM) pictures and the contact angles of working fluids were all taken and compared using the copper sheet.

Heat flux, *q*, is calculated by:

(1) $$q=\left(VI-Q_{\text{hl}}\right)∕A$$

The heat transfer coefficient (HTC), *h*, is calculated by:

(2) $$h=\frac{q}{\Delta T}$$

The uncertainties of *q *and *h *are calculated by:

(3a) $$\frac{U_{q}}{q}=\sqrt{{\left(\frac{U_{V}}{V_{\max}}\right)}^{2}+{\left(\frac{U_{I}}{I_{\max}}\right)}^{2}+{\left(\frac{U_{A}}{A_{\text{max}}}\right)}^{2}+{\left(\frac{U_{Q_{\text{hl}}}}{Q_{\text{hl}}}\right)}^{2}}$$

(3b) $$\frac{U_{h}}{h}=\sqrt{{\left(\frac{U_{q}}{q}\right)}^{2}+{\left(\frac{U_{\Delta T}}{\Delta T}\right)}^{2}}$$

The maximum temperature uncertainty of the thermocouple was 0.2 K. The maximum uncertainties of the power meter and the pressure transducer were 0.5% and 0.2%, respectively. The uncertainty caused by the heating area should be less than 0.5%. The uncertainty of the MHF should be 6.0%, and the maximum uncertainty of the HTC was estimated to be 7.4%.

## Working fluids

Surface-functionalized silica nanoparticles were used to make a kind of stable nanofluid. The functionalization was achieved by grafting silanes to the surface of silica nanoparticles as was introduced by Yang and Liu [26]. Silica nanoparticle powders with an average diameter of about 30 nm and a silane of (3-glycidoxylproyl) trimethyoxysilane (CAS number 2530-83-8) were used for the functionalizing process. The mass ratio of the reacting silane and silica nanoparticles was 0.115. Disperse functionalized nanoparticles into water and then keep the solution at the environmental temperature of 50°C for 12 h. The obtained solution was called functionalized nanofluid.

Functionalized nanoparticles can still keep dispersing well after the nanofluid has been standing for 12 months even at the mass concentration of 10%, and no sedimentation was observed. However, obvious sedimentation of traditional nanofluid (nanofluid consisting of nanoparticles without functionalization) was observed after several days. Traditional nanofluid was also prepared in this study by dispersing and oscillating nanoparticles in water. Silica nanoparticle powders were firstly dispersed into deionized water, and the suspension was then oscillated in an ultrasonic bath for 12 h. The maximum mass concentrations of functionalized nanofluid and traditional nanofluid were both 2.5 wt.% in the present study.

**Figure 3:**
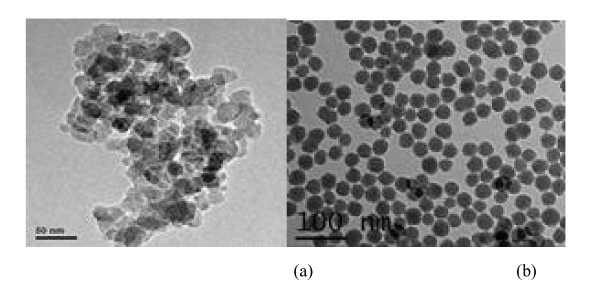
**TEM pictures of nanofluids**. **(a) **Traditional nanofluid and **(b) **functionalized nanofluid.

Figure 3 shows the transmission electron microscope (TEM) pictures of functionalized nanofluid and traditional nanofluid. As is shown, functionalized nanoparticles have no aggregation and can disperse well. The steric stabilization effect and the solubility rule of similarity help nanoparticles disperse uniformly in the base fluid. However, nanoparticles in traditional nanofluid aggregate each other and do not uniformly disperse in the base fluid.

The steric stabilization effect arises from the fact that polymers gathering on the surface of nanoparticles occupy a certain amount of space. If nanoparticles are brought too close together, the space is compressed. An associated repulsive force helps separate nanoparticles from each other and restrains the aggregation of nanoparticles. The grafted silanes mentioned above form the steric stabilization effect and help the nanoparticles disperse uniform in the base fluid.

Besides, to achieve a better and larger solubility of nanoparticles in water, silanes containing polar structures are chosen. Due to the solubility rule of similarity, polar substances are soluble with each other. The polar structure grafted on the surface of the silica nanoparticles increases the solubility of functionalized nanoparticles in water (which is also a polar substance).

Thermophysical properties including the thermal conductivity, the viscosity, and the surface tension of functionalized nanofluid and traditional nanofluid have been introduced by Yang and Liu [26]. For the convenience of readers to get a quantitative view, these parameters are also listed in Tables 1, 2, and 3, respectively.

**Table 1:** Thermal conductivity ratio of two kinds of nanofluids to the base fluid

Mass concentration (wt.%)	Functionalized nanofluid (20°C)	Functionalized nanofluid (40°C)	Functionalized nanofluid (60°C)	Traditional nanofluid (20°C)
0.5	1.01	1.015	1.019	1.014
1	1.0142	1.022	1.027	1.018
1.5	1.0149	1.025	1.032	1.02
2	1.0163	1.028	1.037	1.021
2.5	1.0189	1.033	1.043	1.0267

**Table 2:** Viscosity ratio of two kinds of nanofluids to the base fluid

Mass concentration (wt.%)	Functionalized nanofluid (20°C)	Functionalized nanofluid (40°C)	Functionalized nanofluid (60°C)	Traditional nanofluid (20°C)
0.5	1.083	1.076	1.068	1.025
1	1.13	1.114	1.108	1.052
1.5	1.156	1.139	1.133	1.078
2	1.19	1.172	1.159	1.1
2.5	1.223	1.203	1.189	1.12

**Table 3:** Surface tension ratio of two kinds of nanofluids to the base fluid

Mass concentration (wt.%)	Functionalized nanofluid (20°C)	Functionalized nanofluid (40°C)	Functionalized nanofluid (60°C)	Traditional nanofluid (20°C)
0.5	0.72278	0.71	0.697	0.7858
1	0.71875	0.704	0.686	0.779
1.5	0.71903	0.701	0.684	0.77373
2	0.70833	0.69	0.676	0.765
2.5	0.7125	0.693	0.678	0.759

The density of nanofluids is calculated as:

(4) $$\rho_{\text{nf}}={\left(\frac{1-\omega }{\rho_{\text{w}}}+\frac{\omega }{\rho_{\text{np}}}\right)}^{-1}$$

The specific heat of nanofluids is calculated as:

(5) $$\rho_{\text{nf}}c_{\text{p,nf}}=\rho_{\text{np}}c_{\text{p,np}}\varphi +\rho_{\text{w}}c_{p,\text{w}}\left(1-\varphi \right)$$

The latent heat of nanofluids is the same as that of water.

## Experimental results and discussions

### Surface characteristics of heated surfaces after the experiment using nanofluids

**Figure 4:**
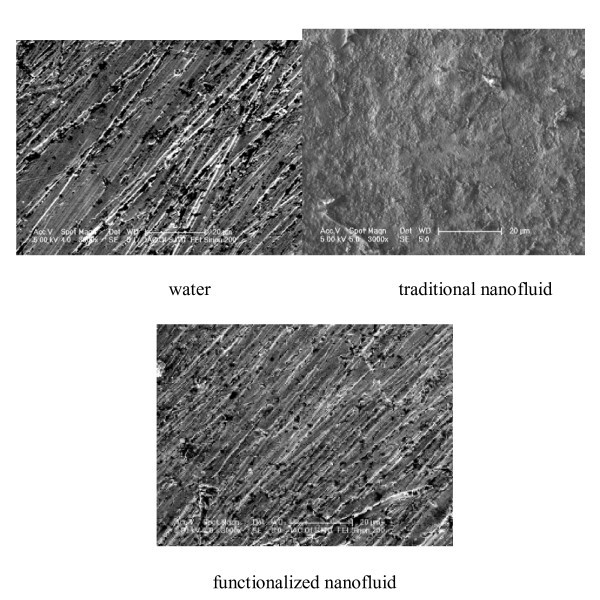
**SEM pictures of heated surfaces**.

Figure 4 shows the SEM pictures of the heated surfaces in the evaporator (copper sheets mentioned in "Experimental apparatus and process") after the test using water, functionalized nanofluid, and traditional nanofluid (called the water-boiled surface, the functionalized nanofluid-boiled surface, and the traditional nanofluid-boiled surface, respectively). The mass concentration of both nanofluids was 1.5 wt.%. The test was carried out at an operating temperature of 40°C. As shown in Figure 4, a deposition layer forms on the traditional nanofluid-boiled surface. However, no deposition layers exist on the functionalized nanofluid-boiled surface. For traditional nanofluid, a part of the reunion bodies of nanoparticles will deposit and be attached to the heated surface. With the evaporating process keep going, more nanoparticles are attached to the heated surface. This results in the forming of the deposition layer, and the layer thickens gradually with the evaporating process.

For functionalized nanofluid, however, nanoparticles in single state cannot form a reunion body; the nanoparticles settled out of the nanofluid can still resolve in the base fluid due to the steric effect and the solubility rule of similarity of the silane. Therefore, no deposition layer exists for functionalized nanofluid. The main purpose of the present study is to investigate the sole effect of the thermophysical properties of nanofluids on the thermal performance of the thermosyphon under the condition that no coating layer exists on the smooth heated surface. This can help eliminate the effect of the surface characteristics.

The SEM pictures of the condensing surfaces in the condenser after the test using functionalized nanofluid and traditional nanofluid were also taken (not plotted in the paper). Different from the surface characteristics of the traditional nanofluid-boiled surface, no deposition layer forms on condensing surfaces for traditional nanofluid.

**Figure 5:**
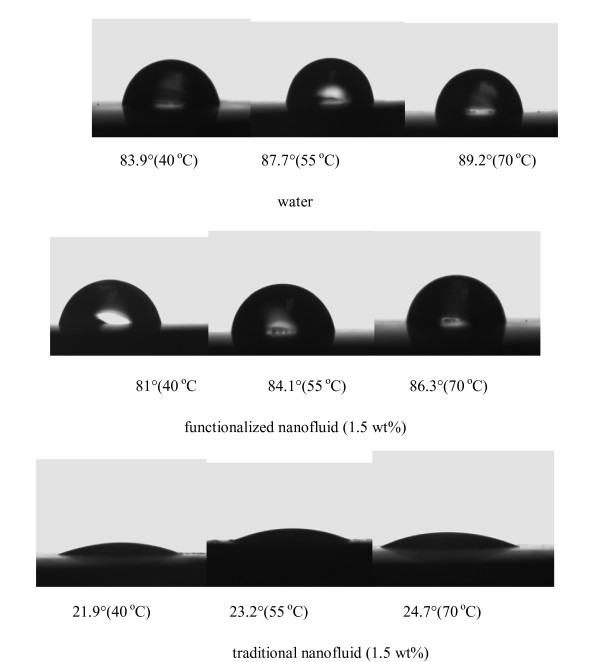
**Contact angle pictures of working fluids**.

Figure 5 shows the contact angle pictures of working fluids on heated surfaces (copper sheets mentioned in Sec. 2"Experimental apparatus and process"). Contact angles were taken using the drop sessile method. Heated surfaces were the ones after the tests using working fluids (with the liquid temperatures of 40°C, 55°C, and 70°C and the mass concentration of 1.5 wt.%). The test environmental temperature was also equivalent to 40°C, 55°C, and 70°C, respectively. As is shown, the contact angle of water on the water-boiled surface is 83.9°, that of functionalized nanofluid on the functionalized nanofluid-boiled surface is 81°, and that of traditional nanofluid on the traditional nanofluid-boiled surface is 21.9° at the temperature of 40°C. The contact angle of functionalized nanofluid only decreases slightly compared with water while that of traditional nanofluid decreases greatly. The deposition layer formed by nanoparticles in traditional nanofluid improves the wettability of nanofluids, which leads to a great reduction of the contact angle. The contact angle shows similar changing trend at other temperatures.

Surface roughness of the heated surface is measured for nanofluids under different mass concentrations given in Table 4. As is shown, the average roughness after the boiling test using the functionalized nanofluid-boiled surface is basically the same as that of water. On the other hand, the surface roughness after the boiling test using traditional nanofluid decreases significantly compared with the water case. The reason should be that the coating layer formed by nanopaticles decreases the surface roughness. The average roughness of the traditional nanofluid-boiled surface keeps nearly the same in the whole concentration range tested.

**Table 4:** Average roughness of the nanofluid-boiled surfaces

Mass concentration	R (nm) of functionalized nanofluid-boiled surface	R (nm) of traditional nanofluid-boiled surface
0	35.1	35.1
0.5 wt.%	37.2	21.4
1.0 wt.%	34.5	23.9
1.5 wt.%	39.3	20.5
2.0 wt.%	40.8	21.1
2.5 wt.%	36.5	19.8

## Heat transfer characteristics of functionalized nanofluid

### Average wall temperatures of the evaporator using functionalized nanofluid

**Figure 6:**
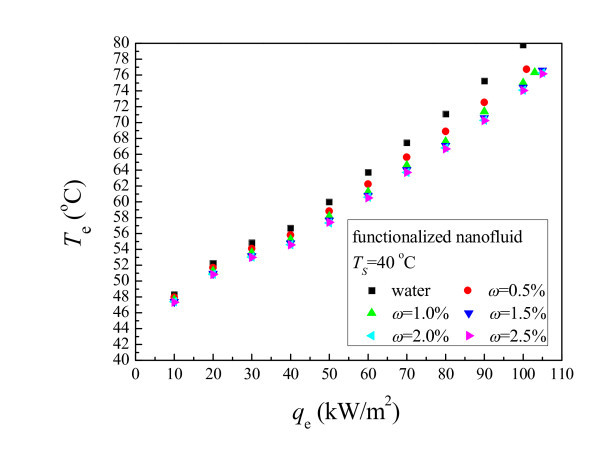
**Average wall temperature of the evaporator using functionalized nanofluid**.

Figure 6 shows the average wall temperatures of the evaporator using functionalized nanofluid at different heat fluxes under the fixed operating temperature of 40°C. As is shown, the average wall temperatures using functionalized nanofluid decreases compared with the water case. They decrease with increasing mass concentrations and the trend slows down gradually. The decrease also increases with increasing the wall heat flux. Functionalized nanofluid enhances the evaporating heat transfer of the thermosyphon. Not plotted in this paper, the average wall temperatures hold the same trend at other operating temperatures.

### The evaporating heat transfer coefficient

**Figure 7:**
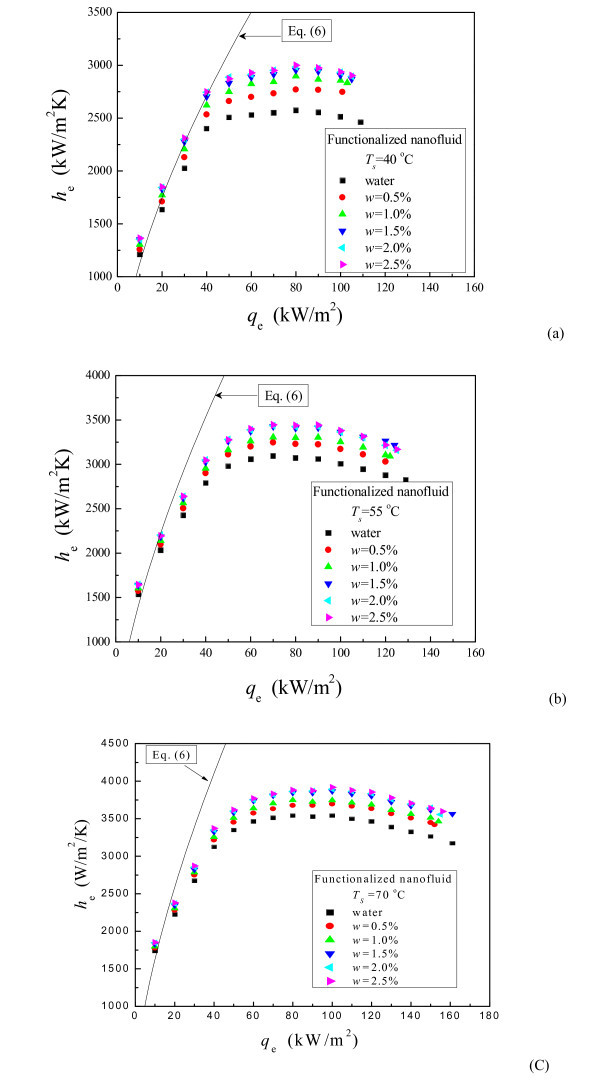
**Effect of mass concentration on the evaporating HTC of functionalized nanofluid**. **(a) ***p *= 7.4 kPa, **(b) ***p *= 15.75 kPa, **(c) ***p *= 31.38 kPa.

Figure 7 illustrates the evaporating heat transfer curves (boiling curves) of functionalized nanofluid in thermosyphon at the operating temperatures of 40°C, 55°C, and 70°C. The mass concentration is 0% (water), 0.5, 1.0, 1.5, 2.0, and 2.5 wt.%, respectively. As is indicated, the heat transfer coefficient (HTC) of functionalized nanofluid increases compared with that of water. Also, it increases with the increase of the mass concentration of nanoparticles, and the increasing trend slows down gradually. There are not much changes for the HTC enhancement ratio when the concentration reaches and exceeds 1.5 wt.%. The evaporating HTC of functionalized nanofluid increases maximally by 17% at the operating temperature of 40°C. In addition, the MHF of functionalized nanofluid is quite close to that of water, which indicates that functionalized nanofluid have nearly no effects on the MHF enhancement.

The calculated HTC curves for water plotted also in Figure 7. Due to the complexity of the heat transfer in thermosyphon, it is hard to find predicting correlations to exactly calculate its evaporating HTC. Therefore, a well-known empirical correlation proposed by Kutateladze, which can well predict the HTC of pool boiling on a smooth metal surface [27], is used to estimate the evaporating CHF in the boiling region.

(6) $$\frac{h}{\lambda }\sqrt{\frac{\sigma }{g\left(\rho_{\text{l}}-\rho_{\text{v}}\right)}}=7.0\times 10^{-4}{\underset{\text{l}}{\mathrm{Pr}}}^{0.35}\times {\left[\frac{q}{\rho_{\text{v}}h_{\text{fg}}\nu_{\text{l}}}\sqrt{\frac{\sigma }{g\left(\rho_{\text{l}}-\rho_{\text{v}}\right)}}\right]}^{0.7}{\left[\frac{p}{\sigma }\sqrt{\frac{\sigma }{g\left(\rho_{\text{l}}-\rho_{\text{v}}\right)}}\right]}^{0.7}$$

As shown in Figure 7, the calculated and experimental values keep good agreement at low and medium heat flux. Then the deviation gradually increases. This is because the heat transfer mode in the evaporator of the thermosyphon is similar to the pool boiling heat transfer at low and medium heat flux, but dry-out area on the heated surface will appear and it increases gradually with increasing the heat flux, leading to the deviation of the present study with the pool boiling heat transfer. With the increase of the dry-out area, the HTC flattens and finally decreases till the dry-out limit happens. Therefore, Equation 6 fails to predict the HTC at high heat flux.

**Figure 8:**
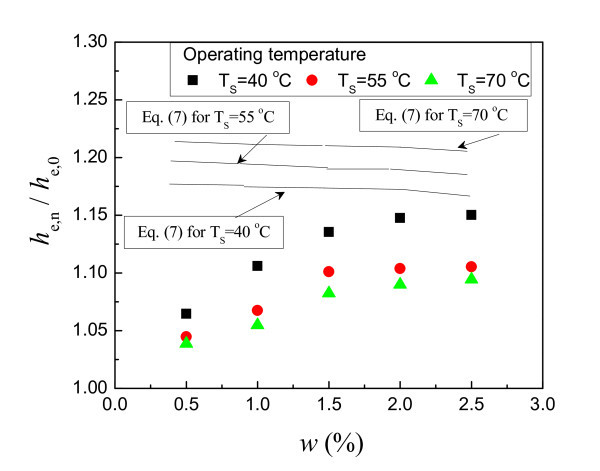
**Effect of mass concentration of functionalized nanofluid on the HTC enhancement ratio**.

Figure 8 indicates the effect of the mass concentration on the evaporating HTC enhancement ratio of functionalized nanofluid (*w *= 1.5 wt.%). Here, the HTC enhancement ratio is an average of ratios in the whole heat flux range tested. As shown in Figure 8, the evaporating HTC enhancement ratio decreases slightly with increasing operating temperature. At the mass concentration of 1.5 wt.%, the evaporating HTC enhancement ratio ranges within 1.12 to 1.16, 1.07 to 1.12, and 1.53 to 1.08, respectively, for the operating temperatures of 40°C, 55°C, and 70°C. The operating temperature has no meaningful influence on the evaporating HTC enhancement ratio.

Besides, the HTC ratio increases with increasing heat flux at all operating temperatures. This effect can be explained by the Brownian motion, and the thermophoresis effect [28]. The thermophoresis effect holds that nanoparticles can diffuse under the effect of a temperature gradient. The diffusion increases with increasing temperature gradient. In the boiling heat transfer, a great temperature gradient exists for the nanofluid near the heated surface. It increases with increasing heat flux and correspondingly increases the diffusion of nanoparticles, and hence the heat transfer is enhanced. Meanwhile, higher temperature leads to stronger Brownian motion, which also enhances the energy transportation. Therefore, the HTC enhancement increases with increasing heat flux.

From Figure 8, it is found that the HTC enhancement results mainly from the sole effect of the thermophysical properties of the nanofluid. According to Equation 6, the HTC and the main thermophysical properties of working fluids hold the following relation at the same heated surface state:

(7) $$\frac{h_{\text{nf}}}{h_{\text{w}}}=\left(\frac{\lambda_{\text{nf}}}{\lambda_{\text{w}}}\right){\left(\frac{\nu_{\text{nf}}}{\nu_{\text{w}}}\right)}^{-0.35}{\left(\frac{\sigma_{\text{nf}}}{\sigma_{\text{w}}}\right)}^{-0.5}$$

The calculated and experimental values hold a deviation of about 15%. This deviation is acceptable due to the experiment error and the inaccuracy of the predicted equation. So it should be considered that Equation 7 can generally predict the HTC enhancement effect caused by the change of the thermophysical properties.

Therefore, the HTC enhancement of the evaporating heat transfer of functionalized nanofluid can be explained by the change of thermophysical properties. Functionalized nanofluid increases the thermal conductivity, the viscosity and decreases the surface tension compared with water. Both the changes of the thermal conductivity and the surface tension increase the HTC while that of the viscosity decreases the HTC. The increasing effect overwhelms the decreasing effect, leading to the HTC enhancement.

However, it should be noted that the experimental data show also an increase trend of the HTC with the increase of the mass concentration. This cannot be explained by Equation 7 since the calculated values are close with each other at different mass concentrations. Also, the HTC enhancement decreases slightly with increasing the operating temperature, which is contrary to the calculated change trend. This shows that Equation 7 can quantitatively calculate the HTC enhancement but is still awkward to qualitatively do that. We will focus on these problems for next-step study.

### Maximum heat flux

There are many empirical and semiempirical equations used for predicting the maximum heat flux (MHF) of a thermosyphon. Imura [29] proposed the following equation in 1983 to predict the MHF of a thermosyphon:

(8) $$q_{\max}=0.16h_{fg}\sqrt[{4}]{\rho_{v}^{2}g\sigma \left(\rho_{\text{l}}-\rho_{\text{v}}\right)}\left\{1-\exp\left[-\left(d∕L_{\text{e}}\right){\left(\rho_{\text{l}}∕\rho_{\text{v}}\right)}^{0.13}\right]\right\}$$

Pioro [30] proposed a similar equation in 1987, which contains the parameter of the contact angle:

(9) $$q_{\max}=0.131h_{fg}\sqrt[{4}]{\rho_{v}^{2}g\sigma \left(\rho_{\text{l}}-\rho_{\text{v}}\right)}{\left\{1-\exp\left[-\left(d∕L_{\text{e}}\right){\left(\rho_{\text{l}}∕\rho_{\text{v}}\right)}^{0.13}\cos^{1.8}\left(\beta -55\right)\right]\right\}}^{0.8}$$

The experimental MHF of functionalized nanofluid and those predicted by Equations 8 and 9 are shown in Table 5. The deviation in Table 5 is defined as

**Table 5:** MHF of water and functionalized nanofluid

	Water			Functionalized nanofluid (1.5 wt.%)	
Operating temperature (°C)	MHF (W/m^2^/k)	Deviation of equation 8	Deviation of equation 9	MHF (W/m^2^/k)	Deviation of equation 9
70	160,863	12.7%	-9.3%	154,602	6.5%
55	128,738	13.0%	-7.7%	124,168	6.8%
40	108,582	2.6%	-12.6%	104,576	-2.6%

(10) $$\text{Dev}=\left(q_{\max,\text{pr}}-q_{\max}\right)∕q_{\max}$$

As shown in Table 5, the maximum deviation of the experimental values and the predicted ones by Equations 8 and 9 for water is smaller than 13.0%. The maximum deviation for functionalized nanofluid is 6.8%. The experimental results indicate that Equations 8 and 9 can also be used to predict the MHF of functionalized nanofluid in a thermosyphon. Since the experimental data keep well with traditional theory, no meaningful nanofluid effect is found for the MHF of functionalized nanofluid.

### Condensing heat transfer characteristics

In general, for Newton fluids, the condensing heat transfer of the falling film along the vertical wall can be estimated by the well-known Nusselt correlation.

(11) $$h_{c}=0.943{\left\{\frac{\rho_{l}g\lambda_{l}^{3}\left(\rho_{l}-\rho_{v}\right)\left[h_{fg}+0.68C_{l}\Delta T_{c}\right]}{\mu_{l}L_{c}\Delta T_{c}}\right\}}^{\frac{1}{4}}$$

**Figure 9:**
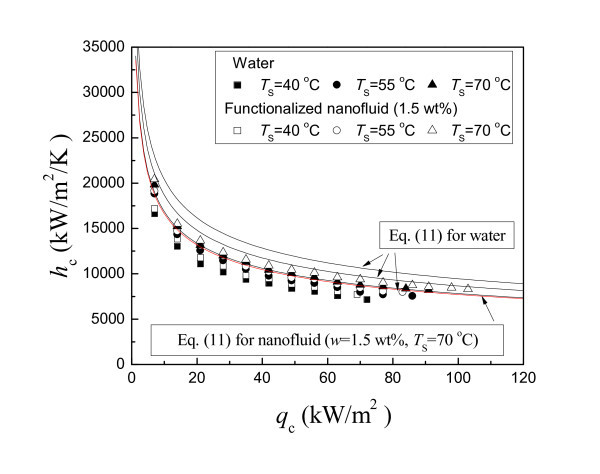
**Condensing HTC curves of functionalized nanofluid**.

Figure 9 shows the experimental data of the condensing HTC for both water and functionalized nanofluid in a thermosyphon at different operating temperatures. The predicted curves of Equation 11 for water and functionalized nanofluid (*w *= 1.5 wt.%) are also shown for comparison. It is found that all experimental data are about 15% less than the calculated values. This is because the flow of the falling film and vapor is countercurrent in the present thermosyphon, and it is reasonable that the experimental data are somewhat less than the calculated values. On the other hand, the condensing heat transfer characteristics of functionalized nanofluid are almost the same as that of water. Adding functionalized nanoparticles into water does not change the condensing heat transfer of the thermosyphon. This experimental result may be well explained by the traditional theory using Equation 11. According to the calculated curves of the condensing HTC of functionalized nanofluid by Equation 11, there exist no meaningful changes between the calculated condensing HTC of functionalized nanofluid and water. The changes of the thermophysical properties have no meaningful effect on the condensing HTC of functionalized nanofluid.

According to the above discussion, functionalized nanofluid can enhance the evaporating HTC of the thermosyphon but has no effect on the MHF and the condensing HTC. The heat transfer characteristics of functionalized nanofluid result mainly from the changes of the thermophysical properties of nanofluids. Therefore, functionalized nanofluid can be considered as an ordinary working fluid and no meaningful nanofluid effect exists for functionalized nanofluid in the thermosyphon.

## Heat transfer characteristics of traditional nanofluid

**Figure 10:**
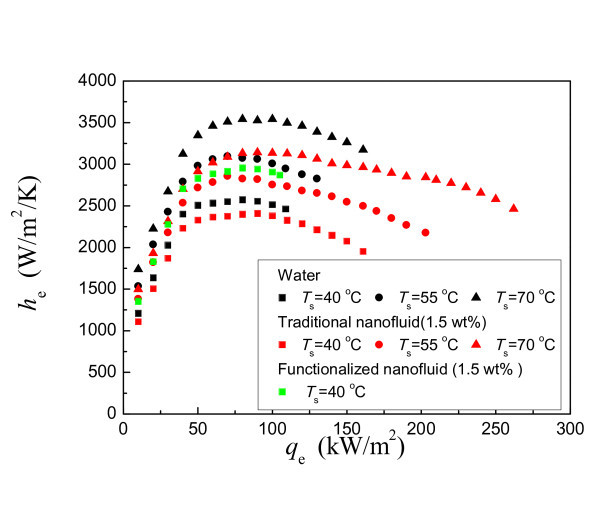
**Evaporating HTC curves of traditional nanofluid**.

Figure 10 indicates the evaporating HTC curves of traditional nanofluid under the three operating temperatures. The mass concentration of traditional nanofluid is fixed at 1.5 wt.%. The HTC curves of water and functionalized nanofluid are plotted also in Figure 10 for comparison. As shown in Figure 10, the evaporating HTC of traditional nanofluid decreases meanly by 7%, 9%, and 11% for the operating temperature of 40°C, 55°C, and 70°C, respectively. The deterioration increases with decreasing operating temperature. On the other hand, the MHF of traditional nanofluid increases obviously with the enhancement ranging within 48% to 63%.

As discussed in the above section, the thermophysical properties of functionalized nanofluid result in HTC enhancement (the data of functionalized nanofluid are also plotted in Figure 10). Traditional nanofluid and functionalized nanofluid have similar trends on the thermophysical properties. Therefore, the thermophysical properties of traditional nanofluid cannot result in the HTC deterioration, and the change of the surface characteristics should mainly attribute to the HTC deterioration.

The deposition layer formed on the heated surface by nanoparticles changes the wettability or the solid-liquid contact angle, the active nucleation site density of the heated surface, and the surface roughness. It also increases the heat resistance of the heated surface. The reduction of the contact angle (the increase in the wettability) and the surface roughness, the increase of the heat resistance all results in the HTC deterioration according to traditional boiling theory, but the deposition layer can also increase the active nucleation site density that can enhance the HTC. The effect of traditional nanofluid on the HTC results from the aggregation of all above factors.

It is hard to estimate quantitatively the number changes of the active nucleation site density, but it should be concluded that the influencing factors leading to the HTC deterioration overwhelm those leading to the HTC enhancement, resulting in the HTC deterioration.

The effect of traditional nanofluid on the condensing heat transfer is the same with that of functionalized nanofluid. Adding nanoparticles does not change the condensing heat transfer. The reason can follow the same explanation for functionalized nanofluid.

For traditional nanofluid, the experimental MHF cannot be predicted by Equations 8 and 9 because the contact angle of traditional nanofluid on the heated surface is over the applicable range of Equations 8 and 9. As is shown in Figure 5, the contact angle of traditional nanofluid in the present study is about 20°; however, Equation 9 can only be used when the contact angle is larger than 55°.

Based on Equation 9, a new equation is arranged which expands the applicable arrangement of the contact angle to 20° to 80°. The new correlation holds a good one to one correspondence for the present MHF data.

(12) $$q_{\max}=0.1424h_{fg}\sqrt[{4}]{\rho_{v}^{2}g\sigma \left(\rho_{\text{l}}-\rho_{\text{v}}\right)}\left\{1-\exp\left[-\left(d∕l_{\text{e}}\right){\left(\rho_{\text{l}}∕\rho_{\text{v}}\right)}^{0.13}\right]\right\}{\left(1+\cos\beta \right)}^{0.8}$$

**Figure 11:**
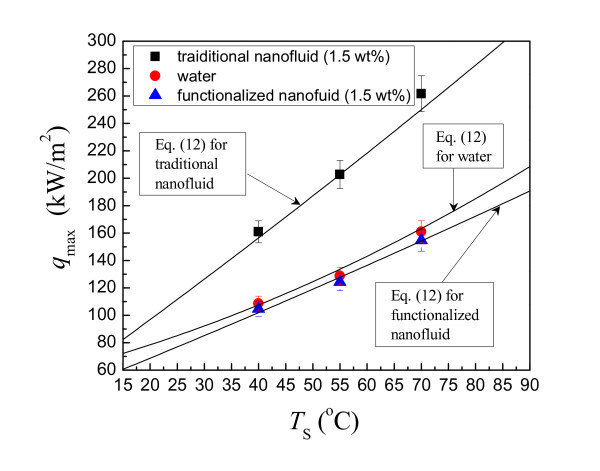
**Comparison of experimental and predicted MHF of working fluids**.

The comparison of the experimental data with Equation 12 is shown in Figure 11. The deviation of Equation 12 lies within 5% for all working fluids, including water, functionalized nanofluid, and traditional nanofluid. Equation 12 confirms that the MHF enhancement of traditional nanofluid results from the decrease of the contact angle. The deposition layer improves its wettability and decrease the contact angle. For functionalized nanofluid, the contact angle only changes very slightly. Therefore, no meaningful enhancement of MHF exists for functionalized nanofluid.

## Conclusions

Surface-functionalized silica nanoparticles were used to prepare a kind of stable nanofluid (called functionalized nanofluid). An experiment was carried out to study the thermal performance of a thermosyphon using water, functionalized nanofluid, and traditional nanofluid (the nanofluid consisting of unfunctionalized nanoparticles) under steady operating pressures. Results are given as:

1. The covalent bonding "Si-O-Si" helps to maintain the steric stabilization effect formed by the grafting silanes which contributes to the long-term stability of nanofluids. Functionalized nanoparticles can still keep dispersing well after the nanofluid has been standing for a long time, and no sedimentation was observed.

2. A deposition layer exists on the heated surface during the experiment using traditional nanofluid; however, no layer exists for functionalized nanofluid. There exist great differences for heat transfer characteristics of functionalized nanofluid and traditional nanofluid. The differences mainly result from the changes of surface characteristics of the heated surfaces but not from the nanofluids themselves.

3. Functionalized nanofluid can enhance the evaporating HTC, while it has generally no effect on the MHF. The HTC enhancement of functionalized nanofluid results mainly from the changes of the thermophysical properties of functionalized nanofluid.

4. Traditional nanofluid deteriorates the evaporating HTC but enhances the MHF. The deposition layer mainly results in the HTC deterioration. The great decrease of the contact angle on the deposition layer corresponds to the MHF enhancement for traditional nanofluid.

5. Functionalized nanofluid and traditional nanofluid both have no effects on the condensing heat transfer of the thermosyphon.

6. In the present study, no meaningful nanofluid effect is found for the heat transfer of nanofluids in the thermosyphon.

## Abbreviations

### Nomenclature

*A*: Heating area (square meters); *c*: Specific heat (joules per kilogram per Kelvin); *d*: Thickness of the inner chamber of thermosyphon (meter); *Dev*: Deviation; *g*: Gravity acceleration (meter per square second); *h*: Heat transfer coefficient (watts per Kelvin per square meter); *h*_fg:_: Latent heat of evaporation (joules per kilogram); *I*: Current (A); *L*: Length (meter); *p*: Pressure (kilopascal); Pr: Prandtl number (-); *Q*: Heat power (watts); *q*: Heat flux (watts per square meter); *T*: Temperature (Kelvin); *ΔT*: Wall superheat (Kelvin); *U*: Uncertainty; *V*: Voltage (Volts); *λ*: Thermal conductivity (watts per meter per Kelvin); *β*: Contact angle (degrees); *w*: Mass concentration; *σ*: Surface tension (Newton per meter); *μ*: Dynamic viscosity (kilograms per meter per second); *ρ*: Density (kilograms per cubic meter)

### Subscripts

C: Condenser; e: Evaporator; hl: Heat loss; l: Liquid; nf: Nanofluid; np: Nanoparticles; max: Maximum; pr: Predicted value; v: Vapor; w: Water.

## Competing interests

The authors declare that they have no competing interests.

## Authors' contributions

XFY carried out the experiment, participated in the theoretical calculation and drafted the manuscript. ZHL carried out the design of the study plan and performed the design of the theoretical model. All authors read and approved the final manuscript.
